# 

**Published:** 1899-10-21

**Authors:** 


					/THE HOSPITAL, "The standard of highest purity." - Lancet. October 21st, 1899.
CADBURY'S U to OADBURY'S
COCOA ))L m COCOA
ABSOLUTELY PURE. ~~~W NO DRUGS OR ALKALIES USED.
No. 681 Vol. XXVII. [KSerLl Saturday,
Oct. 21st, 1899.
j-S^soi^tioiiJenns.j pRICE TWOPENCE.

				

